# 6-Chloro-3-[4-(hex­yloxy)phen­yl]-[1,2,4]triazolo[4,3-*b*]pyridazine

**DOI:** 10.1107/S2414314625005668

**Published:** 2025-07-11

**Authors:** Jasmin Preis, Dieter Schollmeyer, Heiner Detert

**Affiliations:** a University Mainz, Duesbergweg 10-14, 55099 Mainz, Germany; Goethe-Universität Frankfurt, Germany

**Keywords:** crystal structure, heterocycles

## Abstract

Mol­ecules of the title compound, C_17_H_19_ClN_4_O, are essentially planar, the dihedral angles between the planes of the tetra­aza­indene ring system and the benzene ring and between the benzene ring and the hex­yloxy chain being 1.56 (10)° and 5.02 (17)°, respectively. In the crystal, pairs of mol­ecules are connected *via* π–π inter­actions between the phenyl ring and the triazolopyridazine moiety.

## Graphical abstract

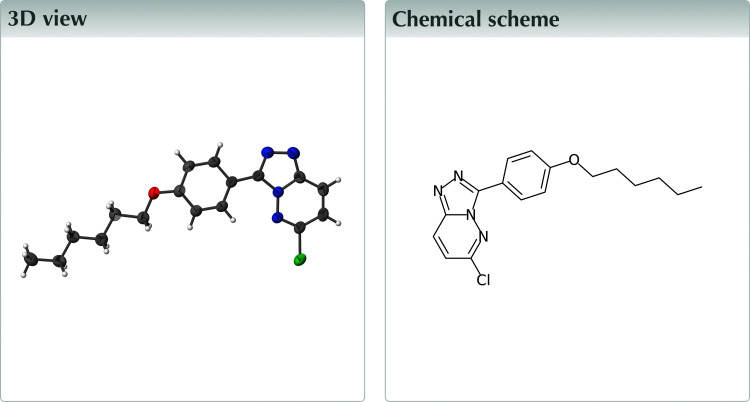

## Structure description

**Figure 1:**
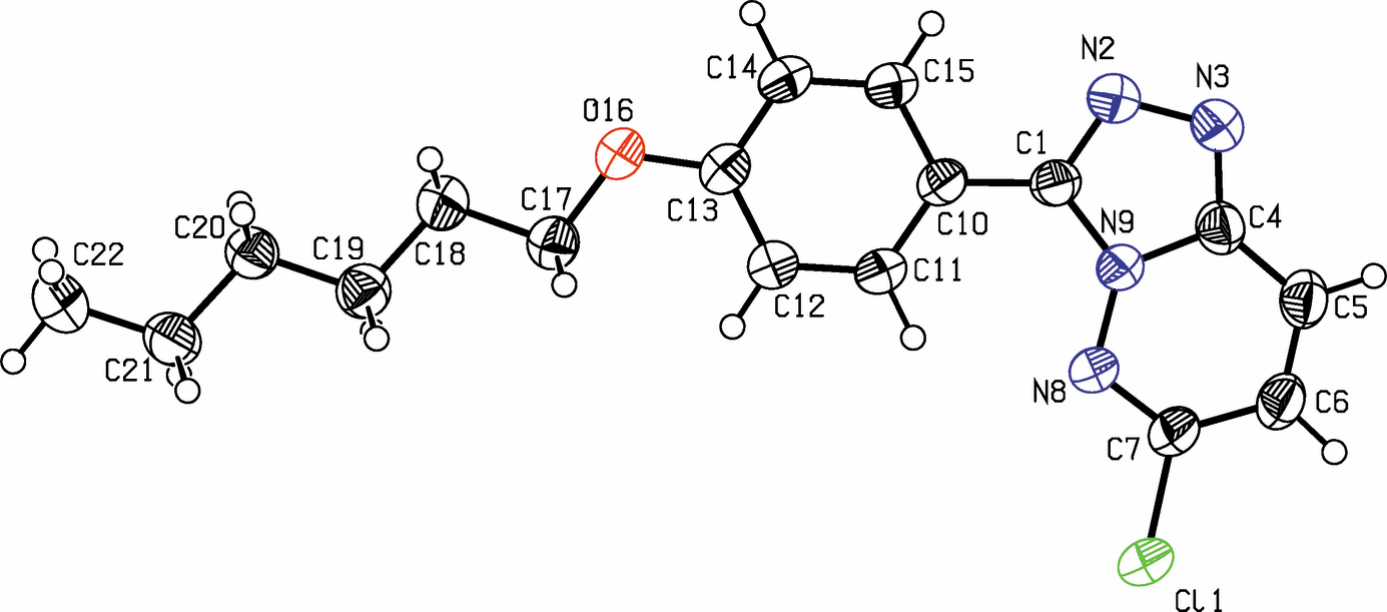
View (Spek, 2009 ▸) of the title compound. Displacement ellipsoids are drawn at the 50% probability level.

**Figure 2:**
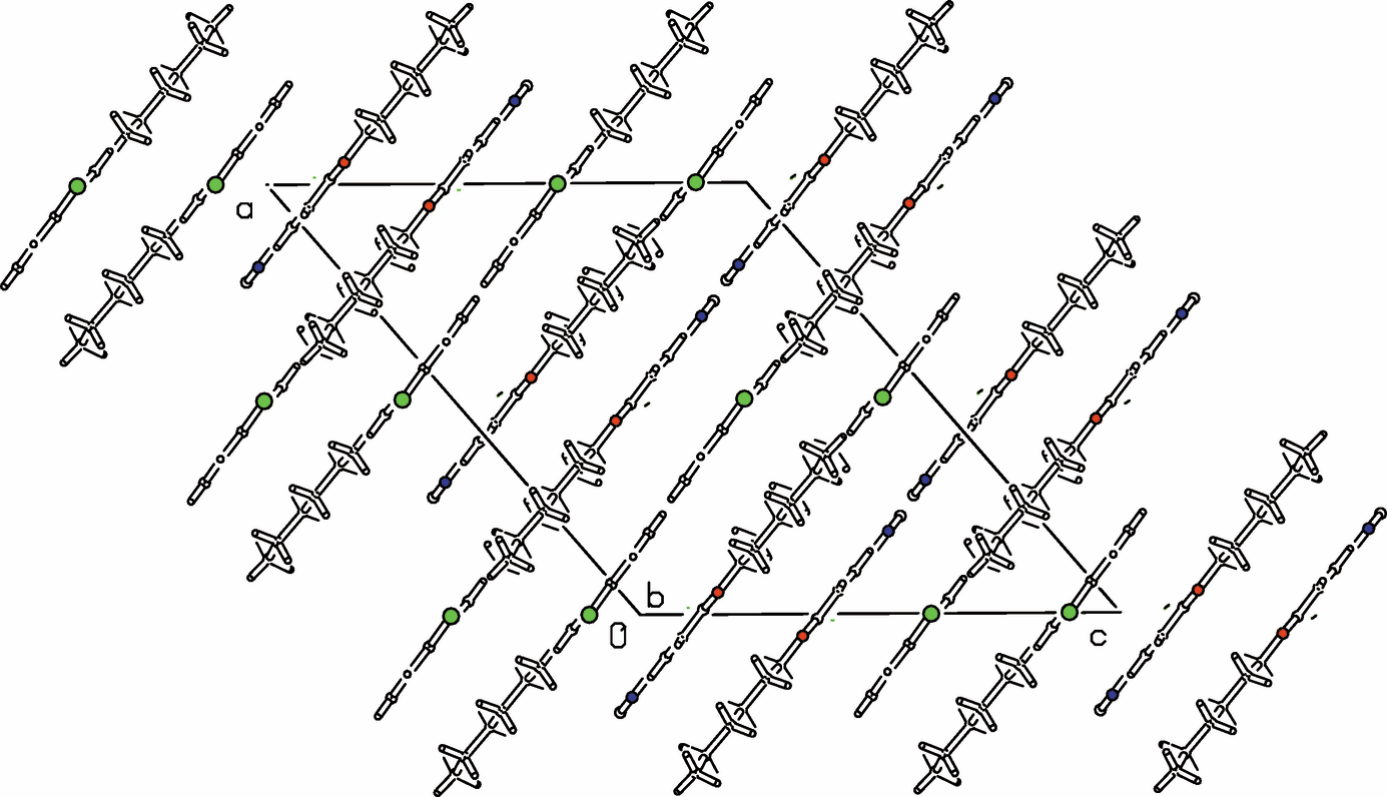
Part of the packing diagram. View along the *b*-axis direction (Spek, 2009 ▸).

The title compound (Fig. 1 ▸) was prepared in preparation for a project on discotic liquid crystals (Jochem *et al.*, 2022 ▸; Rieth *et al.*, 2020 ▸; Tober *et al.*, 2019 ▸). Huisgen and co-workers reported the triazoloannulation to 1,3,5-triazine (Huisgen *et al.*, 1960 ▸), but other chloro­azines are also suitable substrates (Preis *et al.*; 2011 ▸, Schollmeyer & Detert, 2014 ▸). Mol­ecules of the title compound, C_17_H_19_N_4_OCl, are almost completely planar. The tetra­aza­indene framework (C1–N9) is planar (r.m.s. deviation 0.0098 Å). Further minor deviations from planarity are the small dihedral angle of 1.56 (3)° between the planes of tetra­aza­indene and the phenyl ring and of 5.02 (3)° between phenyl ring and the planar hex­yloxy chain in a perfect *all-anti* conformation. A small distance of 3.5056 (13) Å indicates π–π inter­actions between the centroids of the bicyclic unit and of the phenyl ring. These neighbouring mol­ecules are connected *via* a centre of inversion. In the monoclinic unit cell, eight of the planar mol­ecules are arranged in planes parallel to (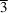
02) (Fig. 2 ▸).

## Synthesis and crystallization

A solution of 5-*p*-hexyl­oxyphenyl­tetra­zole (200 mg) and 3,6-di­chloro­pyrazine (60 mg) in 10 ml xylenes/pyridine (5/1) was slowly heated to reflux and stirred at this temperature for 8 d. The solution was extracted with 1 *N* hydro­chloric acid and brine, solvents were evaporated, and the residue was purified by chromatography on silica using toluene/ethyl acetate 2/1 with 1% tri­ethyl­amine as eluent. Yield: 39 mg (29%) of a brownish, crystalline solid with m.p. = 375 K. The annotation of NMR signals follows IUPAC nomenclature. ^1^H-NMR (400 MHz, CDCl_3_):8.36 (*d*, *J* = 9 Hz, 2 H, 2-H, 6-H ph); 8.09 (*d*, *J* = 9.6 Hz, 1H, 4-H pyridazin), 7.09 (*d*, *J* = 9.6 Hz, 1H, 5-H pyridazin), 7.03 (*d*, *J* = 9 Hz, 2 H, 3-H, 5-H ph), 4.01 (*t*, *J* = 6.6 Hz, 2 H,OCH_2_), 1.83–1.76 (*m*, 2 H, CH_2_), 1.50–1.43 (*m*, 2 H, CH_2_), 1.36–1.31 (*m*, 4 H, CH_2_), 0.90 (*t*, 3 H, CH_3_); ^13^C-NMR (100 MHz, CDCl_3_): 161.0 (C4 ph), 149.0 (C6 pyridazin), 148.0 (C-1 ph), 143.3 (C-3 pyridazin), 129.2 (C-2,6 ph), 126.5 (C4 pyridazin), 121.4 (C-5 pyridazin), 117.6 (C-1 ph), 114.7 (C-3,5 ph), 68.1 (OCH_2_), 31.5, 25.7, 22.6, 14.0 (CH_2_), 14.0 (CH_3_). IR: (ATR): 3045, 2956, 2938, 2918, 2865, 1608, 1538, 1458, 1253, 1057, 830 cm^−1^. FD—MS: 330.1 (*M*^+.^); HR-ESI-MS: found 353.1160, calculated 3351.1145 for *M*+Na^+.^.

## Refinement

Crystal data, data collection and structure refinement details are summarized in Table 1 ▸.

## Supplementary Material

Crystal structure: contains datablock(s) I, global. DOI: 10.1107/S2414314625005668/bt4176sup1.cif

Structure factors: contains datablock(s) I. DOI: 10.1107/S2414314625005668/bt4176Isup2.hkl

Supporting information file. DOI: 10.1107/S2414314625005668/bt4176Isup3.cml

CCDC reference: 2466356

Additional supporting information:  crystallographic information; 3D view; checkCIF report

## Figures and Tables

**Table 1:** Experimental details

Crystal data	
Chemical formula	C_17_H_19_ClN_4_O
*M*_r_	330.81
Crystal system, space group	Monoclinic, *C*2/*c*
Temperature (K)	193
*a*, *b*, *c* (Å)	21.4463 (13), 11.3646 (3), 18.0798 (11)
β (°)	130.696 (2)
*V* (Å^3^)	3341.0 (3)
*Z*	8
Radiation type	Cu *K*α
μ (mm^−1^)	2.10
Crystal size (mm)	0.55 × 0.45 × 0.30

Data collection	
Diffractometer	Enraf–Nonius CAD-4
Absorption correction	ψ scan (*Corinc*; Dräger & Gattow, 1971 ▸)
*T*_min_, *T*_max_	0.842, 0.996
No. of measured, independent and observed [*I* > 2σ(*I*)] reflections	3271, 3163, 2806
*R*_int_	0.059
(sin θ/λ)_max_ (Å^−1^)	0.609

Refinement	
*R*[*F*^2^ > 2σ(*F*^2^)], *wR*(*F*^2^), *S*	0.042, 0.124, 1.04
No. of reflections	3163
No. of parameters	209
H-atom treatment	H-atom parameters constrained
Δρ_max_, Δρ_min_ (e Å^−3^)	0.24, −0.28

Computer programs: *CAD-4 Software* V5 (Enraf–Nonius, 1989 ▸), *Corinc* (Dräger & Gattow, 1971 ▸), *SIR97* (Altomare *et al.*, 1999 ▸), *SHELXL2019/2* (Sheldrick, 2015 ▸) and *PLATON* (Spek, 2009 ▸).

## full crystallographic data

### 6-Chloro-3-[4-(hexyloxy)phenyl]-[1,2,4]triazolo[4,3-b]pyridazine Crystal data

**Table:**

C_17_H_19_ClN_4_O	*D*_x_ = 1.315 Mg m^−^^3^
*M**_r_* = 330.81	Melting point: 375 K
Monoclinic, *C*2/*c*	Cu *K*α radiation, λ = 1.54178 Å
*a* = 21.4463 (13) Å	Cell parameters from 25 reflections
*b* = 11.3646 (3) Å	θ = 65–70°
*c* = 18.0798 (11) Å	µ = 2.10 mm^−^^1^
β = 130.696 (2)°	*T* = 193 K
*V* = 3341.0 (3) Å^3^	Block, light brown
*Z* = 8	0.55 × 0.45 × 0.30 mm
*F*(000) = 1392	

### 6-Chloro-3-[4-(hexyloxy)phenyl]-[1,2,4]triazolo[4,3-b]pyridazine Data collection

**Table:**

Enraf–Nonius CAD-4 diffractometer	2806 reflections with *I* > 2σ(*I*)
Radiation source: rotating anode	*R*_int_ = 0.059
Graphite monochromator	θ_max_ = 70.0°, θ_min_ = 4.6°
ω/2θ scans	*h* = −19→26
Absorption correction: ψ scan (*Corinc*; Dräger & Gattow, 1971)	*k* = 0→13
*T*_min_ = 0.842, *T*_max_ = 0.996	*l* = −22→0
3271 measured reflections	3 standard reflections every 60 min
3163 independent reflections	intensity decay: 2%

### 6-Chloro-3-[4-(hexyloxy)phenyl]-[1,2,4]triazolo[4,3-b]pyridazine Refinement

**Table:**

Refinement on *F*^2^	Primary atom site location: structure-invariant direct methods
Least-squares matrix: full	Hydrogen site location: inferred from neighbouring sites
*R*[*F*^2^ > 2σ(*F*^2^)] = 0.042	H-atom parameters constrained
*wR*(*F*^2^) = 0.124	*w* = 1/[σ^2^(*F*_o_^2^) + (0.0751*P*)^2^ + 1.3637*P*] where *P* = (*F*_o_^2^ + 2*F*_c_^2^)/3
*S* = 1.04	(Δ/σ)_max_ < 0.001
3163 reflections	Δρ_max_ = 0.24 e Å^−^^3^
209 parameters	Δρ_min_ = −0.28 e Å^−^^3^
0 restraints	

### 6-Chloro-3-[4-(hexyloxy)phenyl]-[1,2,4]triazolo[4,3-b]pyridazine Special details

**Table:**

Geometry. All esds (except the esd in the dihedral angle between two l.s. planes) are estimated using the full covariance matrix. The cell esds are taken into account individually in the estimation of esds in distances, angles and torsion angles; correlations between esds in cell parameters are only used when they are defined by crystal symmetry. An approximate (isotropic) treatment of cell esds is used for estimating esds involving l.s. planes.
Refinement. Hydrogen atoms were placed at calculated positions and were refined in the riding-model approximation with C_aromatic_–H = 0.95 Å and with C–H = 0.99 Å for the remaining H atoms, and with *U*_iso_(H) = 1.5 *U*_eq_(C_methyl_) and with *U*_iso_(H) = 1.2 *U*_eq_(C) for the other H atoms.

### 6-Chloro-3-[4-(hexyloxy)phenyl]-[1,2,4]triazolo[4,3-b]pyridazine Fractional atomic coordinates and isotropic or equivalent isotropic displacement parameters (Å^2^)

**Table:**

	*x*	*y*	*z*	*U*_iso_*/*U*_eq_	
Cl1	0.49915 (3)	0.11106 (4)	0.10533 (3)	0.06749 (19)	
C1	0.37217 (9)	0.49223 (14)	−0.07286 (11)	0.0422 (3)	
N2	0.30929 (9)	0.51615 (13)	−0.16533 (10)	0.0541 (4)	
N3	0.27439 (9)	0.41338 (14)	−0.21728 (11)	0.0574 (4)	
C4	0.31738 (10)	0.32689 (15)	−0.15461 (12)	0.0468 (4)	
C5	0.31084 (11)	0.20313 (16)	−0.16525 (13)	0.0510 (4)	
H5	0.268361	0.167534	−0.226405	0.061*	
C6	0.36682 (11)	0.13741 (16)	−0.08582 (13)	0.0522 (4)	
H6	0.365474	0.053973	−0.089820	0.063*	
C7	0.42824 (11)	0.19581 (14)	0.00451 (12)	0.0481 (4)	
N8	0.43661 (8)	0.30881 (11)	0.01962 (9)	0.0428 (3)	
N9	0.37955 (8)	0.37244 (11)	−0.06273 (9)	0.0398 (3)	
C10	0.42205 (9)	0.58130 (13)	0.00276 (11)	0.0408 (3)	
C11	0.48876 (10)	0.55610 (14)	0.09998 (12)	0.0462 (4)	
H11	0.503990	0.476445	0.119914	0.055*	
C12	0.53343 (10)	0.64531 (15)	0.16833 (12)	0.0476 (4)	
H12	0.578976	0.626419	0.234247	0.057*	
C13	0.51163 (10)	0.76159 (14)	0.14048 (12)	0.0441 (4)	
C14	0.44518 (11)	0.78813 (14)	0.04373 (13)	0.0494 (4)	
H14	0.430003	0.867913	0.024251	0.059*	
C15	0.40134 (10)	0.70008 (14)	−0.02385 (12)	0.0476 (4)	
H15	0.356206	0.719750	−0.089757	0.057*	
O16	0.55092 (8)	0.85588 (10)	0.20141 (9)	0.0520 (3)	
C17	0.61958 (11)	0.83485 (15)	0.30264 (12)	0.0496 (4)	
H17A	0.664066	0.793793	0.309993	0.060*	
H17B	0.602617	0.785342	0.331914	0.060*	
C18	0.64901 (11)	0.95325 (16)	0.35216 (12)	0.0517 (4)	
H18A	0.603087	0.994333	0.341648	0.062*	
H18B	0.665797	1.001365	0.322062	0.062*	
C19	0.72129 (11)	0.94299 (16)	0.46149 (13)	0.0513 (4)	
H19A	0.702989	0.900642	0.492264	0.062*	
H19B	0.765200	0.895645	0.471925	0.062*	
C20	0.75647 (10)	1.06144 (16)	0.51114 (12)	0.0489 (4)	
H20A	0.775972	1.102823	0.481346	0.059*	
H20B	0.712101	1.109480	0.499045	0.059*	
C21	0.82697 (11)	1.05264 (17)	0.62029 (13)	0.0558 (4)	
H21A	0.869662	1.000154	0.632441	0.067*	
H21B	0.806465	1.015937	0.650538	0.067*	
C22	0.86625 (13)	1.1704 (2)	0.66969 (15)	0.0689 (6)	
H22A	0.890830	1.204507	0.644107	0.103*	
H22B	0.909034	1.158522	0.740232	0.103*	
H22C	0.824192	1.223861	0.656558	0.103*	

### 6-Chloro-3-[4-(hexyloxy)phenyl]-[1,2,4]triazolo[4,3-b]pyridazine Atomic displacement parameters (Å^2^)

**Table:**

	*U*^11^	*U*^22^	*U*^33^	*U*^12^	*U*^13^	*U*^23^
Cl1	0.0834 (4)	0.0346 (2)	0.0562 (3)	0.00329 (19)	0.0331 (3)	0.00725 (17)
C1	0.0409 (8)	0.0376 (8)	0.0442 (8)	0.0017 (6)	0.0259 (7)	0.0044 (6)
N2	0.0495 (8)	0.0452 (8)	0.0473 (8)	0.0014 (6)	0.0226 (7)	0.0035 (6)
N3	0.0511 (8)	0.0510 (8)	0.0463 (8)	−0.0028 (7)	0.0213 (7)	0.0013 (6)
C4	0.0442 (8)	0.0476 (9)	0.0421 (8)	−0.0065 (7)	0.0254 (7)	−0.0027 (7)
C5	0.0532 (9)	0.0470 (9)	0.0500 (9)	−0.0121 (7)	0.0324 (8)	−0.0089 (7)
C6	0.0608 (10)	0.0381 (8)	0.0559 (10)	−0.0097 (7)	0.0373 (9)	−0.0061 (7)
C7	0.0570 (9)	0.0352 (8)	0.0504 (9)	−0.0023 (7)	0.0342 (8)	0.0021 (7)
N8	0.0469 (7)	0.0346 (7)	0.0421 (7)	0.0004 (5)	0.0270 (6)	0.0035 (5)
N9	0.0404 (6)	0.0351 (6)	0.0398 (7)	−0.0013 (5)	0.0244 (6)	0.0013 (5)
C10	0.0416 (8)	0.0338 (7)	0.0471 (8)	0.0009 (6)	0.0290 (7)	0.0023 (6)
C11	0.0490 (8)	0.0346 (8)	0.0477 (9)	0.0035 (6)	0.0283 (8)	0.0053 (6)
C12	0.0501 (9)	0.0395 (8)	0.0440 (8)	0.0023 (7)	0.0266 (8)	0.0017 (7)
C13	0.0481 (8)	0.0370 (8)	0.0504 (9)	0.0008 (6)	0.0334 (8)	−0.0008 (6)
C14	0.0531 (9)	0.0336 (8)	0.0541 (9)	0.0063 (7)	0.0317 (8)	0.0052 (7)
C15	0.0458 (8)	0.0379 (8)	0.0472 (9)	0.0047 (7)	0.0252 (7)	0.0061 (7)
O16	0.0586 (7)	0.0372 (6)	0.0490 (7)	0.0005 (5)	0.0302 (6)	−0.0034 (5)
C17	0.0540 (9)	0.0458 (9)	0.0483 (9)	0.0007 (7)	0.0331 (8)	−0.0015 (7)
C18	0.0531 (9)	0.0462 (9)	0.0514 (10)	−0.0002 (7)	0.0321 (8)	−0.0034 (7)
C19	0.0529 (9)	0.0499 (10)	0.0521 (9)	−0.0004 (8)	0.0346 (8)	0.0001 (8)
C20	0.0504 (9)	0.0496 (10)	0.0451 (9)	0.0020 (7)	0.0304 (8)	0.0015 (7)
C21	0.0532 (9)	0.0597 (11)	0.0466 (9)	0.0050 (8)	0.0290 (8)	0.0039 (8)
C22	0.0638 (12)	0.0718 (14)	0.0470 (10)	−0.0048 (10)	0.0255 (9)	−0.0034 (9)

### 6-Chloro-3-[4-(hexyloxy)phenyl]-[1,2,4]triazolo[4,3-b]pyridazine Geometric parameters (Å, º)

**Table:**

Cl1—C7	1.7169 (17)	C14—C15	1.371 (2)
C1—N2	1.322 (2)	C14—H14	0.9500
C1—N9	1.369 (2)	C15—H15	0.9500
C1—C10	1.460 (2)	O16—C17	1.435 (2)
N2—N3	1.375 (2)	C17—C18	1.508 (2)
N3—C4	1.318 (2)	C17—H17A	0.9900
C4—N9	1.384 (2)	C17—H17B	0.9900
C4—C5	1.414 (2)	C18—C19	1.526 (2)
C5—C6	1.346 (3)	C18—H18A	0.9900
C5—H5	0.9500	C18—H18B	0.9900
C6—C7	1.427 (2)	C19—C20	1.518 (2)
C6—H6	0.9500	C19—H19A	0.9900
C7—N8	1.301 (2)	C19—H19B	0.9900
N8—N9	1.3642 (18)	C20—C21	1.516 (2)
C10—C11	1.393 (2)	C20—H20A	0.9900
C10—C15	1.404 (2)	C20—H20B	0.9900
C11—C12	1.389 (2)	C21—C22	1.522 (3)
C11—H11	0.9500	C21—H21A	0.9900
C12—C13	1.383 (2)	C21—H21B	0.9900
C12—H12	0.9500	C22—H22A	0.9800
C13—O16	1.364 (2)	C22—H22B	0.9800
C13—C14	1.390 (2)	C22—H22C	0.9800
			
N2—C1—N9	107.90 (14)	C10—C15—H15	119.5
N2—C1—C10	124.21 (14)	C13—O16—C17	118.61 (13)
N9—C1—C10	127.88 (14)	O16—C17—C18	107.02 (14)
C1—N2—N3	109.95 (14)	O16—C17—H17A	110.3
C4—N3—N2	106.39 (13)	C18—C17—H17A	110.3
N3—C4—N9	109.83 (15)	O16—C17—H17B	110.3
N3—C4—C5	132.29 (16)	C18—C17—H17B	110.3
N9—C4—C5	117.88 (15)	H17A—C17—H17B	108.6
C6—C5—C4	117.79 (16)	C17—C18—C19	112.29 (15)
C6—C5—H5	121.1	C17—C18—H18A	109.1
C4—C5—H5	121.1	C19—C18—H18A	109.1
C5—C6—C7	118.57 (16)	C17—C18—H18B	109.1
C5—C6—H6	120.7	C19—C18—H18B	109.1
C7—C6—H6	120.7	H18A—C18—H18B	107.9
N8—C7—C6	126.68 (16)	C20—C19—C18	113.06 (15)
N8—C7—Cl1	115.18 (13)	C20—C19—H19A	109.0
C6—C7—Cl1	118.14 (13)	C18—C19—H19A	109.0
C7—N8—N9	113.04 (13)	C20—C19—H19B	109.0
N8—N9—C1	128.05 (13)	C18—C19—H19B	109.0
N8—N9—C4	126.02 (13)	H19A—C19—H19B	107.8
C1—N9—C4	105.92 (13)	C21—C20—C19	113.61 (15)
C11—C10—C15	117.73 (15)	C21—C20—H20A	108.8
C11—C10—C1	124.19 (14)	C19—C20—H20A	108.8
C15—C10—C1	118.08 (14)	C21—C20—H20B	108.8
C12—C11—C10	121.19 (15)	C19—C20—H20B	108.8
C12—C11—H11	119.4	H20A—C20—H20B	107.7
C10—C11—H11	119.4	C20—C21—C22	113.99 (16)
C13—C12—C11	120.03 (15)	C20—C21—H21A	108.8
C13—C12—H12	120.0	C22—C21—H21A	108.8
C11—C12—H12	120.0	C20—C21—H21B	108.8
O16—C13—C12	124.93 (15)	C22—C21—H21B	108.8
O16—C13—C14	115.63 (14)	H21A—C21—H21B	107.7
C12—C13—C14	119.44 (16)	C21—C22—H22A	109.5
C15—C14—C13	120.52 (15)	C21—C22—H22B	109.5
C15—C14—H14	119.7	H22A—C22—H22B	109.5
C13—C14—H14	119.7	C21—C22—H22C	109.5
C14—C15—C10	121.08 (15)	H22A—C22—H22C	109.5
C14—C15—H15	119.5	H22B—C22—H22C	109.5
			
N9—C1—N2—N3	0.05 (19)	N2—C1—C10—C11	−179.38 (16)
C10—C1—N2—N3	−178.86 (14)	N9—C1—C10—C11	1.9 (2)
C1—N2—N3—C4	−0.1 (2)	N2—C1—C10—C15	1.0 (2)
N2—N3—C4—N9	0.12 (19)	N9—C1—C10—C15	−177.68 (15)
N2—N3—C4—C5	−179.79 (18)	C15—C10—C11—C12	−0.1 (2)
N3—C4—C5—C6	178.23 (18)	C1—C10—C11—C12	−179.74 (15)
N9—C4—C5—C6	−1.7 (2)	C10—C11—C12—C13	0.4 (3)
C4—C5—C6—C7	1.2 (2)	C11—C12—C13—O16	179.81 (15)
C5—C6—C7—N8	0.2 (3)	C11—C12—C13—C14	−0.4 (3)
C5—C6—C7—Cl1	179.87 (14)	O16—C13—C14—C15	179.88 (15)
C6—C7—N8—N9	−1.0 (2)	C12—C13—C14—C15	0.0 (3)
Cl1—C7—N8—N9	179.28 (10)	C13—C14—C15—C10	0.3 (3)
C7—N8—N9—C1	−178.30 (15)	C11—C10—C15—C14	−0.2 (2)
C7—N8—N9—C4	0.5 (2)	C1—C10—C15—C14	179.43 (15)
N2—C1—N9—N8	179.00 (14)	C12—C13—O16—C17	−0.9 (2)
C10—C1—N9—N8	−2.1 (3)	C14—C13—O16—C17	179.32 (14)
N2—C1—N9—C4	0.02 (17)	C13—O16—C17—C18	−178.57 (13)
C10—C1—N9—C4	178.88 (15)	O16—C17—C18—C19	178.74 (13)
N3—C4—N9—N8	−179.09 (14)	C17—C18—C19—C20	175.09 (14)
C5—C4—N9—N8	0.8 (2)	C18—C19—C20—C21	178.53 (15)
N3—C4—N9—C1	−0.09 (18)	C19—C20—C21—C22	176.26 (17)
C5—C4—N9—C1	179.84 (15)		
